# Human MAP Tau Based Targeted Cytolytic Fusion Proteins

**DOI:** 10.3390/biomedicines5030036

**Published:** 2017-06-27

**Authors:** Olusiji A. Akinrinmade, Sandra Jordaan, Dmitrij Hristodorov, Radoslav Mladenov, Neelakshi Mungra, Shivan Chetty, Stefan Barth

**Affiliations:** 1 South African Research Chair in Cancer Biotechnology, Institute of Infectious Disease and Molecular Medicine (IDM), Department of Integrative Biomedical Sciences, Faculty of Health Sciences, University of Cape Town, Kapstadt 7700, South Africa; alex.akinrinmadex@gmail.com (O.A.A.); sandrajordaanibms@gmail.com (S.J.); MNGNEE002@myuct.ac.za (N.M.); shivan.chetty@gmail.com (S.C.); 2 Fraunhofer Institute for Molecular Biology and Applied Ecology, 52074 Aachen, Germany; dmitrij.hristodorov@rwth-aachen.de (D.H.); radoslav.mladenov@rwth-aachen.de (R.M.)

**Keywords:** human cytolytic fusion proteins, immunotherapy, microtubule associated protein tau (MAP), cancer, inflammatory diseases

## Abstract

Some of the most promising small molecule toxins used to generate antibody drug conjugates (ADCs) include anti-mitotic agents (e.g., auristatin and its derivatives) which are designed to attack cancerous cells at their most vulnerable state during mitosis. We were interested in identifying a human cystostatic protein eventually showing comparable activities and allowing the generation of corresponding targeted fully human cytolytic fusion proteins. Recently, we identified the human microtubule associated protein tau (MAP tau), which binds specifically to tubulin and modulates the stability of microtubules, thereby blocking mitosis and presumably vesicular transport. By binding and stabilizing polymerized microtubule filaments, MAP tau-based fusion proteins skew microtubule dynamics towards cell cycle arrest and apoptosis. This biological activity makes rapidly proliferating cells (e.g., cancer and inflammatory cells) an excellent target for MAP tau-based targeted treatments. Their superior selectivity for proliferating cells confers additional selectivity towards upregulated tumor-associated antigens at their surface, thereby preventing off-target related toxicity against normal cells bearing tumor-associated antigens at physiologically normal to low levels. In this review, we highlight recent findings on MAP tau-based targeted cytolytic fusion proteins reported in preclinical immunotherapeutic studies.

## 1. Introduction

**Figure 1:**
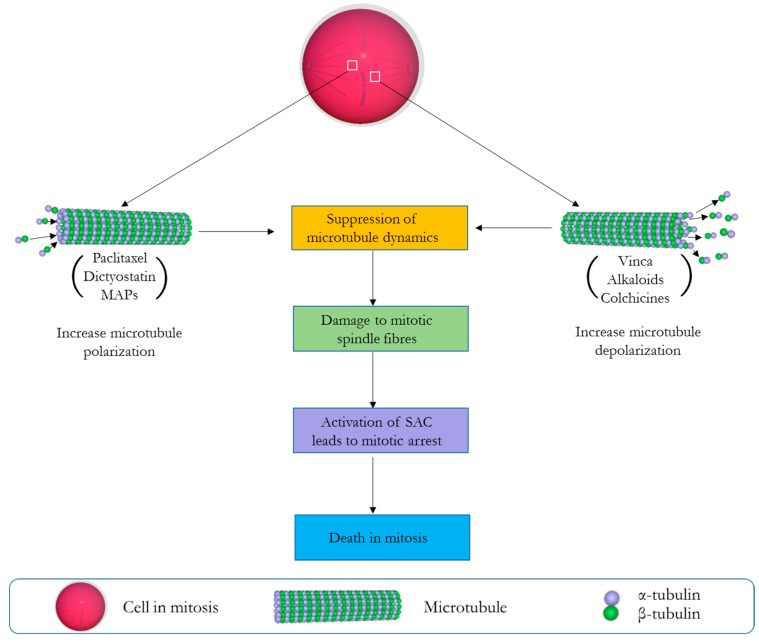
Mechanistic scenario of events leading to mitotic cell death (MCD) by microtubule targeting drugs. Microtubule stabilizing agents (e.g., paclitaxel, dictyostain, microtubule associated proteins—MAPs) promote the formation of microtubule bundles that cannot disassemble, while microtubule destabilizing agents (e.g., vinca alkaloids, colchicines) prevent the formation of the mitotic spindle by inhibiting tubulin polymerization. Both result in the activation of the spindle assembly checkpoint (SAC), eventually leading to mitotic arrest and apoptosis.

Of all pharmacological agents developed for the treatment of cancer, anti-mitotic drugs have until now remained the most successful [1]. All such drugs are largely defined by their profound ability to compromise cellular dynamics during the crucial period of mitotic cell division, and by so doing initiate apoptosis or autophagy-related cell death [2]. As a reason of their success, traditional anti-mitotic agents like the vinca alkaloids have remained an integral part of anti-cancer agents available to clinicians since their first use about seven decades ago [3]. Their clinical effectiveness has encouraged the developments of newer therapeutics against several cellular and molecular targets involved in different stages of mitosis (e.g., microtubules, kinases, motor proteins, and multi-protein complexes) [2]. Importantly, those targeting the microtubules have been regarded as the most clinically effective [4]. They work by either stabilizing or destabilizing the polymerization of microtubule, therein disrupting the organization of mitotic spindle necessary for the completion of the M phase of the cell cycle [5]. Normally, microtubules springing from the opposite poles of a dividing cell during metaphase will allow spindle fibres to make productive attachment to the kinetochores and by so doing establish connections necessary for the precise arrangement of chromosomes along the equatorial plate. This is particularly dependent on the dynamic nature of microtubules through its polymerization (grow) and depolymerization (shorten) cycles which allows the microtubule to search the cytoplasm for the kinetochores [6,7]. Importantly, the precise alignment of chromosomes at the equatorial plate is a pre-requisite to allow spindle assembly checkpoint—SAC (a cell cycle regulator) to permit the commencement of anaphase. This is because any abnormal alignment or the absence of a single chromosome at the equatorial plate will prevent the cell from completing mitosis and eventually lead to the induction of apoptosis [8]. Most microtubule targeting drugs (stabilizing or destabilizing) exploit this delicate moment of mitotic cell division to exert their cytotoxic activity, often by the common mechanism of suppressing microtubule dynamics and the chronic activation of the SAC, which halts cell-division in a prolonged event that signals the induction of apoptosis (Figure 1) [9]. Anti-mitotics like the vinca alkaloids (vinflunine, vincristine, vinorelbine, vindesine, and eribulin), cryptophycins, halichondrins, estramustine, colchicines, and combretastatins are examples of drugs known to depolymerize/destabilize microtubules by binding to various β-tubulin sites [5,10,11]. On the other hand, taxanes paclitaxel (Taxol), laulimalide, dictyostatin, the epothilones, etc. are examples of microtubule stabilizing compounds [12,13] known to enhance tubulin polymerization/stabilization and prevent the ability of microtubules to shorten or separate sister chromatids [10,14]. Clinically, the vinca alkaloids and taxanes have been integrated in combination chemotherapy regimens for the treatment of breast cancer, ovarian cancer, non-small-cell lung carcinoma, and haematological malignancies [15]. Based on their popularity, semi-synthetic and synthetic strategies have been used to produce analogues of these agents for a wider therapeutic application [16]. This is because most of these agents are scarce and only found in minute amounts in their natural sources (mostly marine organisms and plants), herewith slowing down clinical development [17]. Examples includes auristatin E (AE) and monomethyl auristatin E (MMAE), which are derivatives of the tubulin polymerisation inhibitor dolastatin 10 [18]. Interestingly, these analogues are highly effective and have shown more cytotoxicity than their natural counterparts with preclinical studies showing AE to be 200 times more potent than vinblastine [19]. The therapeutic effectiveness of MMAE at the cytotoxic domain of brentuximab vedotin has also resulted in its approval by the US Food and Drug Administration (FDA) for the treatment of refractory Hodgkin lymphoma and systemic anaplastic large-cell lymphoma [20,21]. Unfortunately, as this is not often the case with other microtubule binding agents (especially the synthetic derivatives of taxanes, faced with several clinical drawbacks [17]), we were interested in identifying a human anti-mitotic protein eventually showing comparable activities and allowing the generation of corresponding fully human cytolytic fusion proteins.

We identified microtubule associated protein tau (MAP tau), which belongs to a family of proteins (microtubule associated proteins, MAPs) endowed with the ability to bind tubulin and modulate the stability of microtubules. Different classes of MAPs occur in different cell types, and are known to have specific functions. Of interest to scientists are MAPs that can stabilize microtubules, including MAP2, MAP4, and MAP tau [22]. They act primarily by binding to microtubules and by promoting the formation of crosslinks between tubulin units. By promoting tubulin assembly, the rate of depolymerization is reduced and an overall stabilization effect is observed. In so doing, these proteins promote the rapid formation of the mitotic spindle, which allows for the movement of chromosomes to opposite poles during mitosis [23]. As the enzymatic phosphorylation of MAPs is a tightly regulated system for controlling microtubule dynamics and ensuring a successful cell division [24], we postulated that the presence of non-phosphorylated MAPs would considerably slow down mitosis and force the cell to undergo apoptosis [18]. The implementation of MAP tau as an effector protein in fully humanized antibody-based immunotherapy was not accidental, but based on its vital role in regulating microtubule behaviour. Thus, the first MAP tau-based fusion protein was engineered, bearing specificity towards the human epidermal growth factor (EGF) receptor [4]. In this review, we provide a summary of the biology of MAP tau, including data from preclinical studies incorporating the selective cytotoxicity of MAP tau towards proliferating cells for the development of targeted human cytolytic fusion proteins.

## 2. Microtubule Associated Protein (MAP) Tau: Discovery and Structure

Tau proteins are found in many animal species, including *Drosophila* [25], *rodents* [26], *goats* [27], *goldfish* [28], and *humans* [29]. Discovered in 1975 by Weingarten et al. by co-purification with tubulin [30], MAP tau was recognized as a protein factor essential for microtubule assembly [31]. It is most abundantly expressed in the neurons of the human brain [12] and results from a single tau gene present on chromosome 17 [32]. However, evidence shows that MAP tau expression is not solely restricted to neuronal cells. Differential trace levels of MAP tau are also detectable in the heart, muscle, testis, lung, and pancreatic tissues [33,34].

Interestingly, when subjected to electrophoresis on sodium dodecyl sulfate polyacrylamide gels, it was found that purified tau consists of four to six polypeptides of molecular weights ranging from 55 to 62 kDa [35]. Subsequent discoveries then led to the confirmation that alternative splicing of tau mRNA indeed results in the formation of six isoforms in adult mammalian brain tissue [36]. In addition to these isoforms, several discoveries suggest the expression of a high molecular weight isoform in the peripheral nervous system [37]. This isoform—termed “Big tau”—is characterized by the presence of an additional large exon (4a) and a lower ability to self-aggregate [38,39]. Experiments carried out on the longest-lived rodents indicated a protective role of this high molecular weight isoform [40]. Taken altogether, these facts capitalize on the heterogeneous distribution of tau within cells and between cell populations. However, they all exhibit a similar structure, differing only in the number of microtubule-binding domains, as well as in the number of N-terminal inserts. In general, tau proteins consist of a C-terminal region that encompasses a proline-rich domain which is capable of interacting with microtubules [41]. On the other hand, the N-terminal domain consists of repeats of amino acids which do not bind to tubulin. By projecting from the microtubule surface, tau’s N-terminal domains mediate the interaction between microtubules and the plasma membrane, thereby promoting neuronal development [42].

### 2.1. MAP Tau Function

The primary role of MAP tau revolves around its ability to stabilize and promote the polymerization of microtubules. Indeed, a study conducted in 1992 demonstrated that the addition of tau to pure tubulin solutions culminates in an increase in the rate of growth of microtubules [43]. Such an observation is also accompanied by hindrance of progression into the shrinking phase of microtubule dynamics. Through new technological approaches (e.g., cryo-electron microscopy), it was determined that tau binds in a longitudinal fashion to microtubules, and that such binding results in the bridging of tubulin interfaces [44].

Several in vitro experiments were carried out to characterize the defining role of MAP tau in neuronal development. By selectively inhibiting tau expression in cell culture, it was found that neuronal elongation was severely affected [45]. While these results demonstrated an indispensable role of tau in the neuron, other studies begged to differ. For example, a tau knockout study in mice did not report any kind of abnormalities in brain development; microtubule stability, or neuronal growth [46]. This study showed that other nervous systems MAPs might be able to compensate for tau deficiency. A surprising discovery was the upregulation of microtubule associated protein 1A (MAP1A) in the brain of mice lacking the tau gene [46]. However, the exact mechanism by which an increase in MAP1A level may compensate for lost tau function is unclear, since the primary structure or microtubule interaction motifs of MAP1A are reportedly different to those of tau. Indeed, tau’s functions are directly related to its peculiar structure. For instance, tau requires its internal repeat domain (IRD) and the flanking regions on either side of the IRD to promote the formation of bundles when assembling microtubules. The regions between repeats 1 and 2 are also important, and regulate tau affinity towards microtubule binding [47]. MAPs with homologous binding domains to tau include MAP2 and MAP4. MAP2 has a homologous tau repeat domain near its carboxyl terminus and are often found with three repeats, although a four repeat MAP2c isoform has now been discovered [47]. MAP2 has two binding sites on a tubulin dimer; it can bind to the same site as MAP4 and tau, and another site which is uncompetitive by other MAPs [48]. MAP4—unlike MAP2 and tau—is ubiquitously found in cells outside the nervous system [49], and possess homologous repeats to tau by which they interact and stabilize microtubules.

Overall, it can be speculated that each tau isoform has different functions based on structural composition. This claim is warranted by the varying spatial and temporal expression of MAP tau variants. For instance, only one tau isoform is prominently expressed during fetal stages of development, while all six isoforms are usually present during adulthood [50]. Additionally, fetal tau undergoes a higher level of phosphorylation as compared to adult tau [51]. Together, these studies provide evidence of the cooperative role of isoforms and phosphorylation in regulating microtubule dynamics.

### 2.2. Pathologies Associated with MAP Tau

To ensure the normal biological function of tau, a regulatory network of kinases and phosphatases has proved to be highly indispensable. A KXGS motif found within tau’s repeat domain [52] is known to be the site of phosphorylation, to which any abnormalities in the degree of phosphorylation can have serious impacts on an organism’s well-being. Clinically, aggregation of filamentous tau proteins is a distinctive feature of various neurodegenerative diseases, collectively termed as tauopathies [53]. Some examples include Pick’s disease, progressive supranuclear palsy, as well as Alzheimer’s disease (AD) [54]. AD remains one of the most common occurrences of such pathology. It is characterized by an excessive phosphorylation at Ser396 [55] and the accumulation of extracellular plaques and intraneuronal neurofibrillary tangles consisting primarily of β-amyloid peptides and hyper-phosphorylated MAP tau, respectively [56]. The pathology of such a disease may be in part due to the loss of tau’s ability to bind to tubulin. Microtubule destabilisation thereby follows and causes a serious threat to neuronal integrity. By removing the vital phosphorylation sites of tau, we made a bona fide attempt towards preventing any possible hyper-phosphorylation of MAP tau-based fusion proteins. Theoretically, it is also not realistic that these fusion proteins will cross a healthy blood–brain-barrier (BBB). This could only be possible in patients already suffering from neurodegenerative diseases (e.g., Multiple sclerosis (MS) or AD) and having a leaky BBB. Furthermore, the selective nature of human cytolytic fusion proteins towards their target receptors helps prevent the accumulation of MAP tau in the human brain.

### 2.3. Map Tau-Based Targeted Human Cytolytic Fusion Proteins

**Figure 2:**
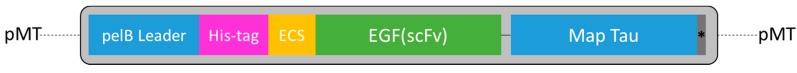
Schematic representation of the expression cassette for the recombinant epidermal growth factor (EGF)–MAP human cytolytic fusion protein. The open reading frame (ORF) was expressed in *E. coli* BL21(DE3) using protocol for periplasmic stress expression in the presence of compatible solutes and includes an N-terminal pectate lyase B (pelB) leader sequence from *Erwinia carotovora* which directs the fusion protein to the periplasmic space, a His_10_ tag to facilitate purification, and an enterokinase cleavage site (ECS) to allow proteolytic separation of the fusion construct from the upstream production sequence. * = C-terminal nuclear localization signal sequence (NLS).

For the first time, we demonstrated the selective delivery and cytotoxicity of a constitutively active human microtubule stabilizing protein towards cancer cells by reporting a new drug format incorporating the human MAP tau protein in the fashion of a targeted human cytotylic fusion protein. The expression construct for this fusion protein contained the genetic information for the human MAP tau (isoform 3) protein mutated at two phosphorylation sites and genetically fused to the human anti- epidermal growth factor receptor (EGFR) single chain fragment variable (scFv). This double mutation (S156A and S204A) allows MAP tau to bind microtubules without negative regulation, and by so doing, interrupt their dynamic “grow” and “shorten” behaviour, ultimately leading to mitotic arrest and induction of apoptosis. To allow nuclear enrichment of the internalised protein, we inserted a nuclear localization signal sequence (NLS) by adding the sequence (5′-CCCAAAAAAAAAAGGAAAGTG-3′) derived from the SV40 T-antigen to the C-terminus of the open reading frame (ORF). A schematic representation of this expression construct is depicted in Figure 2 below.

Based on our hypothesis that a constitutively active anti-EGFR-MAP tau fusion protein would trigger apoptosis in a proliferation-dependent manner, we tested the specificity of the fusion protein in in vitro and in vivo experiments (for clarity, MAP tau in a fusion construct will henceforth be described as MAP). Specific binding of the EGF(scFv)–MAP tau construct to EGFR-overexpressing pancreatic cancer cell line (L3.6pl) was revealed by flow cytometry with the absence of non-specific binding to EGFR^−^ HEK293 cells. Cellular cytotoxicity assays revealed that the EGF(scFv)–MAP fusion protein had an IC_50_ value of ~1 µM against L3.6pl cells with no cytotoxicity towards EGFR^−^ HEK293 cell line in vitro, which importantly demonstrates the selective cytotoxicity of human MAP tau towards its targeted ligand. Next, we further evaluated the proliferation-dependent cytotoxicity of EGF(scFv)–MAP by halting cell division with a pre-titrated concentration (100 pM for 24 h) of paclitaxel known to arrest mitosis. Importantly, this mitosis blocking reagent is confirmed (paclitaxel, at above concentration) not to induce apoptosis in cell lines [57]. Findings from the study revealed that no cytotoxic activity was evident for EGF(scFv)–MAP in non-proliferating cells in comparison to the Pseudomonas exotoxin A (ETA’) based immunotoxin 425(scFv)–ETA’ which was highly cytotoxic (IC_50_ ~1 nM) to L3.6pl cells arrested at the G1 growth phase. Furthermore, apoptosis and tubulin polymerisation assays showed EGF(scFv)–MAP to induce apoptosis by promoting microtubule polymerisation. Taking advantage of the popularity of xenograft models to screen and evaluate new anti-cancer agents for their clinical potential [58], we investigated the efficacy of EGF–MAP in BALB/C nu/nu mice subcutaneously injected with Katushka-2 (far-red fluorescent protein) transfected L3.6pl cells. An anti-tumor treatment cycle of 4 mg·kg^−1^ EGF(scFv)–MAP intravenously administered to mice at 11 days after tumor challenge resulted in a significant reduction in tumor growth when compared to the control group that received phosphate-buffered saline (PBS) [18]. More recently, subsequent evaluation of several MAP tau-based human cytolytic fusion proteins have been described and are summarized in Table 1 below.

As depicted in the table above, most of the MAP tau-based fusion proteins confer improved IC_50_ values when compared to the IC_50_ value of EGF(scFv)–MAP from its first use, even though they were constructed on the same Map tau ORF described earlier. This difference could mainly be linked to the different binding affinity of the targeting moiety and/or internalization frequency of target cell receptors towards the MAP-tau bound ligand, hence the different concentration of the protein available to confer its cytolytic activity. Additionally, all the MAP-tau based fusion proteins recorded above were favourably well tolerated in vivo at an intravenous dose of 4 mg·kg^−1^. Interestingly, this treatment schedule was sufficient to significantly inhibit tumor growth when compared with the control group. It is also important to note that the cytotoxicity conferred by this new class of therapeutics is comparably able to compete with other effector proteins—especially those of human origin. The anti-CD30 single-chain antibody fragment Ki4(scFv) fused in frame to MAP tau for treatment of CD30^+^ malignancies such as Hodgkin lymphoma (HL) and systemic anaplastic large cell lymphoma (sALCL) was shown to induce apoptosis in rapidly proliferating L540cy, L428, and Karpas 299 cells in a dose-dependent manner with IC_50_ values of ~53 nmol/L, which is comparable to that obtained (IC_50_ 36 nmol/L) for the Gb_R201K-Ki4(scFv), a granzyme B-derived mutant. Additionally, an anti-EpCAM(scFv)–MAP fusion protein showed promising results with IC_50_ values of 43 and 67 nmol/L against L3.6pl and A431 EpCAM^+^ cancer cell lines, which is also comparably better when compared with that obtained for anti-EpCAM(scFv)–ETA’ (409 nmol/L on L3.6pl and 91 nmol/L on A431 [60]. As described earlier, it is important to note that differences in IC_50_ values are attributed to different target antigens as well as their differences in expression on different cells and cell lines. The highly promising serpin B9-resistant Gb_R201K–Ki4(scFv) construct has also been documented in literature to possess single-digit IC_50_ values, with picomolar IC_50_ values also published in literature for the bacterial toxin ETA’. On the other hand, the cytotoxicity of MAP tau-based fusion proteins can still be greatly improved by the insertion of adapter sequences that facilitate the translocation of the effector molecule from the endosome to the cytosol [65]. This is because most endogenously secreted human molecules are entirely dependent on transport vesicles to move from one cellular location to another, unlike the bacterial enzymes which possess a natural evolutionarily adapted membrane translocation domain which allows a higher concentration of the effector enzyme per target substrate in the cytosol. Hetzel et al. in 2008 reported the significant contribution of small cleavable adapter sequences to enhancing the cytotoxicity of human cytolytic fusion proteins. Indeed, the use of these adapter sequences is a major aim in our future MAP tau-based preclinical studies [60].

Another mechanistic study into the apoptosis pathway induced by compromising microtubules dynamics by MAP tau has also offered a significant advantage to better understanding their underlying mechanism of action (MOA). Amoury and colleagues recently highlighted the possibility of a completely caspase-independent pathway for a fusion protein consisting of the high affinity CSPG4-specific single-chain antibody fragment fused to MAP tau when used on MDA-MB-231 cells. Investigation of the cellular apoptotic pathways activated by αCSPG4(scFv)–MAP in MDA-MB-231 cells revealed no signal for caspase-3 and poly(ADP-ribose) polymerase-1(PARP-1) in treated cells, and a moderate activation of caspase 9 which was not sufficient to match the cytotoxic readouts of MAP tau [62]. A closer look confirmed the significant activation of the mitochondrial EndoG apoptotic pathway by αCSPG4(scFv)-MAP which is similar to that reported for taxanes. Our understanding of the apoptosis signalling pathways after induction of mitotic arrest is crucial to better optimizing human MAP tau-based proteins for clinical use. Further studies are therefore required to fully demonstrate how MAP tau-based human cytolytic fusion proteins induce cell death in targeted cells.

Finally, potential clinical applications of MAP tau-based human cytolytic fusion proteins do not end with their anti-cancer properties. Several other diseases, including atherosclerosis, rheumatoid arthritis, psoriasis, idiopathic pulmonary fibrosis, scleroderma, cirrhosis of the liver, etc. are reportedly driven by cells undergoing excessive or dysregulated proliferation [66]. It is of general agreement that atherogenic proliferation of smooth muscle cells in the inner-most layer (intima) of the arterial wall is a principal event that occurs during the early stages of the pathogenesis of atherosclerosis, which would result in the formation of new connective tissue and intracellular and extracellular lipid deposit [66,67]. Additionally, the chronic inflammatory skin disease psoriasis is characterized by the hyper-proliferation and aberrant differentiation of keratinocytes [68]. In a recent publication by Hristodorov, Mladenov et al. [63], the proliferative state of disease-causing pro-inflammatory macrophages (M1) has also been reported in tissue sections from inflamed skins. This discovery became of significant importance in shedding more light on the current debate of whether macrophages proliferate in situ. It also allowed the evaluation and documentation of the first MAP tau-based fusion protein—H22(scFv)–MAP—for the treatment of diseases mediated by M1 pro-inflammatory macrophages. In the study, authors documented the proliferation-dependent cytotoxicity of H22(scFv)-MAP towards the pro-monocytic cell line HL-60. Interestingly, H22(scFv)-MAP could not kill peritoneum-derived murine macrophages and human macrophages derived from peripheral blood mononuclear cells (PBMCs) because they are known to stop proliferating when isolated and cultivated in vitro. On top of that, ex vivo data demonstrated that H22(scFv)–MAP efficiently recognizes CD64^+^ leukemic blasts, leading to their elimination, while it spares the homeostasis of healthy CD64^+^ PBMC. The same study showed the ability of the human cytolytic fusion protein to kill leukemic cells independently of the target receptor profile, which suggests the high potency of MAP tau as cytostatic agent [64]. In the context of leukaemia, similar results on the efficacy of MAP tau were obtained when evaluating CD89 as a target for immunotherapy [69]. Taken together, these findings confirm the versatility and the therapeutic suitability of MAP-based immunotherapeutic agents for the treatment of a wide range of indications, including immunological diseases.

## 3. Conclusions and Future Remarks

On a much broader landscape, the introduction of MAP tau has culminated in the creation of a wider portfolio of targeted human cytolytic fusion proteins, in addition to human granzymes and RNases. As described above, MAP tau-based fusion proteins have demonstrated the preclinical potential and ability to compromise mitosis and induce apoptosis in proliferating target cells. MAP tau-based fusion proteins offer two levels of protection by (a) target cell surface antigen specificity and (b) killing of proliferating cells. This would prevent healthy cells expressing tumor or disease markers at physiological levels from being affected. On the other hand, it is important to mention that studies aimed at evaluating the retention ability of MAP tau in target cells are still needed and crucial to the eventual success of MAP tau-based fusion proteins. This is because cells in the G1 and S-phase of cell division possess the potential to repopulate cleared fractions once the drug clears, since they are generally not affected by anti-mitotic drugs. The ability of paclitaxel to linger for about a week in tumor cells and by so doing confer longer cytotoxic effect in comparison to the ~13 h half-life of newer drugs has been linked to its popularity and success [70]. Further studies along this line might thus help to improve the effectiveness of MAP tau. However, MAP tau also has its own advantage when compared with other anti-cancer or anti-mitotic agents. It is well tolerated in vivo, as seen in the above findings, which could allow the administration of repeated doses. The human origin of MAP tau is expected to confer a low immunogenic response when compared with other treatment modalities. With the active removal of vital phosphorylation sites, lower off-target toxicities should be associated with this emerging novel class of treatment.

## Figures and Tables

**Table 1:** Cytotoxicity of microtubule associated protein (MAP)-tau based human cytolytic fusion proteins to different cell lines.

Construct	Cell Line	Disease Model	IC_50_ Value (nmol/L)	Reference
EGF(scFv)–MAP	L3.6pl	Pancreas Carcinoma	1000	[18]
	PC-3	Prostrate Carcinoma	2500	
	C4-2	Prostrate Carcinoma	2800	
Ki-4(scFv)–MAP	L540cy	HL and sALCL	53	[59]
	L428	HL and sALCL	135	
	Karpas 299	HL and sALCL	220	
Anti-EpCAM(scFv)–MAP	L3.6pl	Pancreas Carcinoma	43	[60]
	A431	Epidermoid Carcinoma	67	
	22Rv1	Prostate Carcinoma	677	
	C4-2	Prostate Carcinoma	161	
	SU86.86	Pancreas Carcinoma	333	
scFv35–MAP	FL-OH1	Rhabdomyosarcoma	900	[61]
	RD	Rhabdomyosarcoma	950	
αCSPG4(scFv)–MAP	MDA-MB-231	TNBC	219	[62]
	Hs 578T	TNBC	480	
H22(scFv)–MAP	HL-60 cells	M1 macrophage-mediated diseases	0.04	[63]
	CD64^+^ leukemic blasts	AML/CML		[64]

AML: acute myeloid leukemia; CML: chronic myelomonocytic leukemia; HL: Hodgkin lymphoma; sALCL: systemic anaplastic large cell lymphoma; scFv: single chain fragment variable; TNBC: triple negative breast cancer; EpCAM: Epithelial cell adhesion molecule; CSPG4: Chondroitin sulfate proteoglycan 4.
